# Newspaper coverage of biobanks

**DOI:** 10.7717/peerj.500

**Published:** 2014-07-31

**Authors:** Ubaka Ogbogu, Maeghan Toews, Adam Ollenberger, Pascal Borry, Helene Nobile, Manuela Bergmann, Timothy Caulfield

**Affiliations:** 1 Faculties of Law and Pharmacy & Pharmaceutical Sciences, University of Alberta, Edmonton, Alberta, Canada; 2 Health Law Institute, Faculty of Law, University of Alberta, Edmonton, Alberta, Canada; 3 Department of Public Health and Primary Care, KU Leuven, Leuven, Belgium; 4 Department of Epidemiology, German Institute of Human Nutrition, Potsdam-Rehbruecke, Nuthetal, Germany; 5 Faculty of Law and School of Public Health, University of Alberta, Edmonton, Alberta, Canada

**Keywords:** Biobanks, Media representations, Public perceptions, ELSI, Consent, Privacy, Evidence-based policy

## Abstract

**Background.** Biobanks are an important research resource that provides researchers with biological samples, tools and data, but have also been associated with a range of ethical, legal and policy issues and concerns. Although there have been studies examining the views of different stakeholders, such as donors, researchers and the general public, the media portrayal of biobanks has been absent from this body of research. This study therefore examines how biobanking has been represented in major print newspapers from Australia, Canada, the United Kingdom and the United States to identify the issues and concerns surrounding biobanks that have featured most prominently in the print media discourse.

**Methods.** Using Factiva, articles published in major broadsheet newspapers in Canada, the US, the UK, and Australia were identified using specified search terms. The final sample size consisted of 163 articles.

**Results.** Majority of articles mentioned or discussed the benefits of biobanking, with medical research being the most prevalent benefit mentioned. Fewer articles discussed risks associated with biobanking. Researchers were the group of people most quoted in the articles, followed by biobank employees. Biobanking was portrayed as mostly neutral or positive, with few articles portraying biobanking in a negative manner.

**Conclusion.** Reporting on biobanks in the print media heavily favours discussions of related benefits over risks. Members of the scientific research community appear to be a primary source of this positive tone. Under-reporting of risks and a downtrend in reporting on legal and regulatory issues suggests that the print media views such matters as less newsworthy than perceived benefits of biobanking.

## Introduction

Biobanks are research platforms that hold human biological samples, such as DNA, and associated datasets, such as health and demographic information. Biobanks are an important research resource that provides researchers with biological samples, tools and data for a variety of purposes, including the study of disease, and the analysis of complex interactions between genes and the environment (Bemmels, Wolf & Van Ness, 2012). As a result of their perceived value, jurisdictions throughout the world have invested heavily in the creation of large-scale biobanking initiatives.

However, biobanks are also associated with a range of ethical, legal and policy issues and concerns, including the much-debated question of whether donors should be re-contacted and re-consented before their stored biological materials and associated health data are utilized for research projects that were not included in the terms of their original consent to donation (Caulfield & Kaye, 2009; Caulfield, Rachul & Nelson, 2012; Helgesson, 2012; Knoppers, Zawati & Kirby, 2012, 400–403). Keeping donor information secure and confidential is another concern, especially in light of risks associated with open access movements (Lowrance & Collins, 2007), advances in data re-identification techniques (Lowrance & Collins, 2007; Homer et al., 2008; Gymrek et al., 2013), and worries that inappropriate disclosure may lead to genetic discrimination (Rothstein, 2007; Kreiner & Irion, 2013). In addition, there is an ongoing debate in the academic community regarding the value, sustainability, feasibility and affordability of biobanks and biobanking initiatives (Caulfield et al., 2014; Allen et al., 2014; Hogarth & Sullivan, 2013; Bracken et al., 2013; Allen et al., 2012; Collins, 2012; Manolio et al., 2012; Vaught et al., 2011; Davies, 2011; Kaiser, 2009; The Lancet, 2009; Palmer, 2007; The Lancet, 2007; Frank et al., 2006; Manolio, Bailey-Wilson & Collins, 2006; Wallace, 2005; Smith et al., 2005; Collins, 2004; Louis, 2003; Wallace, 2003; Barbour, 2003; Wallace, 2002).

A number of research studies have provided valuable insights on how these issues and concerns are viewed by affected groups, including donors, researchers and the general public (Thiel et al., 2014; Caulfield, Rachul & Nelson, 2012; Kaufman et al., 2009). For example, a recent survey of Alberta residents found that while a majority of respondents would prefer to be asked for permission to use their stored biological samples for future research only once, a similar majority believed they retained ongoing control over the fate of their samples (Caulfield, Rachul & Nelson, 2012). In another study, 90% of respondents surveyed expressed concerns about researchers’ access to their clinical health information held by biobanks and the possibility that information arising from research studies on their stored biological samples could be used against them (Kaufman et al., 2009).

While these studies have helped inform academic and policy reflection, some perspectives are missing. One such perspective is how the popular press views and represents biobanking initiatives, policies and controversies. Research has shown that media representations play a role in many areas of biomedical research, by helping to inform and shape the views of various stakeholders (Petersen, 2002; Seale, 2003; Nisbet & Mooney, 2007; Bubela et al., 2009; Caulfield & Rachul, 2011). Media representations also have an impact on policy debates and development, and on public discourse (Davidson, Hunt & Kitzinger, 2003; Caulfield, Bubela & Murdoch, 2008; Bubela et al., 2009), especially as relates to framing issues and “facts” for consideration (Nisbet, Brossard & Kroepsch, 2003; Holliman, 2004; Kitzinger & Williams, 2005). While we need to be careful not to overstate the impact of the media in this context, the fact remains that the media plays some role in informing public opinion and policy debates on both advances in and social issues arising from biomedical research and other scientific activities.

With the foregoing in mind, this study examines how biobanks have been represented in newspapers from Australia, Canada, the United Kingdom and the United States. The primary aim of the study is to identify the issues and concerns surrounding biobanks that have featured most prominently in the print media discourse. Even though print newspapers are on the decline as a source of news (The PEW Research Center, 2011; Riesch, 2011), newspapers remain influential, are still a primary source for approximately one third of the population (Wellcome Trust, 2012) and can inform the content of other sources of science and health news.

## Methods

We searched Factiva for articles published in major broadsheet newspapers (based on circulation) in Canada, US, UK and Australia (see Table 1 for a list of included newspapers, by country of publication). Our primary search term was “biobank”. The term was selected based on a recent empirical study that suggests it is the correct and most commonly used descriptor of the type of research platform that is the focus of this study (Hewitt & Watson, 2013). Specifically, the study established that the term “biobank” was first used to describe “human population based collections”, but has since been commonly and “increasingly broadly” used to describe “human disease-based collections as well as …animal and other types of biological collections”, along with associated demographic or health data, that are “managed according to professional standards” (Hewitt & Watson, 2013). The search was conducted on July 16, 2013, and was not restricted by date. The search yielded 191 articles.

**Table 1:** Newspapers, by country.

**Canada**	The Globe and Mail; The National Post; Toronto Star; Montreal Gazette; Vancouver Sun
**UK**	The Daily Telegraph; The Financial Times; The Guardian; The Times (London)
**USA**	New York Times; The Wall Street Journal; USA Today; The Washington Post
**Australia**	Sydney Morning Herald; The Age; The Australian

To allow for a more robust sample for the intended analysis, we conducted a second search on the same date using a number of secondary search terms, in the following combinations: (a) “bank” + (“medical” or “health”); (b) “tissue” + (“medical” or “health”); and (c) “research” + (“medical” or “health”). These search combinations were based on a cursory review of words appearing in articles collected in the initial search or consisted of terms we considered analogous to, or likely to appear in articles discussing the primary search term. Our initial searches using these combinations yielded over 1,000 articles. To reduce this number to a manageable number, we restricted the search by date to between July 16, 2006 and the search date (i.e., seven years from the search date). The cut-off date coincides roughly with the period when the term “biobank” began to gain prominence in academic discourse (Hewitt & Watson, 2013), and limiting the search to this date ensured that our additional sample was more relevant to the usage of the term “biobank” in newspaper reports. Restricting the first search (for “biobank”) in a similar manner would have eliminated 94 articles from our original yield. Since the term “biobank” was our primary and preferred search term, we decided against restricting that search by date, and to include all articles collected from that search in our final sample.

Next, we screened the articles and excluded duplicates and articles that discussed the search terms tangentially, such as financial reports and advertisements seeking donations to biobanks. This process yielded a final sample of 163 articles.

Two raters, including one of the authors of this study (AO), independently analyzed a portion of the final sample (Rater A analyzed 81 articles, and Rater B analyzed 82) using a coding frame developed iteratively by initially coding 20 articles and retooling it to avoid vague or irrelevant variables (see “Supplemental Information 1” for the coding frame). The coding frame explored the following variables: information regarding the articles and newspapers in which they appeared, discussion or mentions of the search terms, location of biobanks mentioned or discussed in the articles, discussions regarding funding sources, biological materials, disease conditions, patients or donors, information regarding benefits and risks, raters’ impressions of the manner in which biobanks or biobanking is portrayed in the articles, and mentions or discussions of legal, policy or regulatory issues relating to biobanking. The coding frame was designed to allow for rating of multiples, such as where a quoted individual is identified in the news report as both a researcher and clinician or where an article mentions more than one disease or condition. Where necessary, multiples were analyzed manually and included in the final calculations for associated variables.

To assess the reliability of the results, a third rater (MT), who was not involved in the research design, coded a random selection of 10% of the articles. Inter-rater reliability was assessed using the Landis & Koch (1977) benchmark scale for strength of agreement denoted by kappa. Kappa scores ranging from moderate to almost perfect agreement were obtained for the tested or reported variables (see Table 2). Disagreements between coders on the moderate scores (four out of seventeen of the tested variables) were resolved by consensus. No reanalysis was required for the moderate scoring variables as the third rater agreed with the interpretation adopted by the two original raters.

**Table 2:** Kappa scores and agreement rating for inter-rater tested variables. Landis & Koch (1977) benchmark scale for strength of agreement denoted by kappa: <0.00 = *poor*, 0.00–0.20 = *slight*, 0.21–0.40 = *fair*, 0.41–0.60 = *moderate*, 0.61–0.80 = *substantial,* 0.81–1.00 = *almost perfect*.

Tested variable	Kappa statistic	Strength of agreement
What, if any, is the primary biological material represented in the article?	0.732	Substantial
Was a patient/donor quoted in the discussion of biobanking?	1.000	Almost perfect
Was a researcher quoted in the discussion of biobanking?	0.766	Substantial
Was a biobank representative quoted in the discussion of biobanking?	0.775	Substantial
Are benefits of biobanking mentioned?	0.769	Substantial
If so, how are the benefits framed?	0.423	Moderate
What is the main benefit discussed?	0.713	Substantial
Does the article mention or discuss health benefits?	1.000	Almost perfect
Does the article mention or discuss discrimination in the health insurance context?	1.000	Almost perfect
Does the article mention or discuss discrimination in other contexts?	0.640	Substantial
Does the article mention or discuss risks?	0.870	Almost perfect
If so, how are the risks framed?	0.645	Substantial
What is the main risk discussed?	0.606	Moderate
How is biobanking portrayed in the article generally?	0.509	Moderate
If portrayed positively, why?	0.735	Substantial
If portrayed negatively, why?	0.700	Substantial
Are legal, policy, or regulatory issues relating to biobanking mentioned?	0.444	Moderate

Finally, results were analyzed in SPSS 21 and by manual review of text obtained from the articles. To determine statistical relevance and whether the observed sample of nominal scale conformed to an expected distribution, Pearson’s Chi-Square (*χ*^2^) tests were performed. Test values are presented where the observed deviation from the null hypothesis (no difference in categories) is significant (*p* < 0.05).

## Results

For ease of reference, some of the results discussed below (frequencies only) are presented Appendix S1.

### Coverage and authorship

Of the 163 newspaper articles we reviewed, 85 (52.1%) appeared in the four UK newspapers included in the study, 30 (18.4%) each in the four US and five Canadian newspapers, and 18 (11%) in the three Australian newspapers. The Guardian (UK) published more stories (31 or 19% of articles) than other newspapers in the study, followed by the Financial Times (UK) (21 or 12.9% of articles), The Times (London) (20 or 12.3% of articles), the New York Times (15 or 9.2% of articles) and the Montreal Gazette (12 or 7.4% of articles). At least one article about biobanking was published in each of the years we studied (except for 1999–2001), with the highest number of articles appearing in 2007 (29, or 17.8%). 51 or 31.3% of the articles appeared in the News section of the newspapers (a similar number/percentage did not specify a section), 12 or 7.4% in the Science section, and 8 or 4.9% in the Health or Lifestyle section. The majority of the articles were rated as news articles (78 or 47.9%), investigative reports or news analysis (53 or 32.5%). Other categories include editorials or opinion pieces (14 or 8.6%) and letters (6 or 3.7%). 89 articles (54.6%) were written by a health or science reporter, 41 (25.2%) by other reporters, and 5 articles (3.1%) were expert commentaries. The articles included in our final sample were written by 123 different authors, and with the exception of Clive Cookson, a reporter for the Financial Times, who contributed 10 articles, and Roger Highfield, a reporter for The Daily Telegraph, who wrote 8 articles, no individual author contributed more than six articles (see Appendix S2 for a list of articles analyzed).

The highest number of articles per year were featured in 2007 and 2012. Almost half of the articles from 2007 that mentioned a specific biobank mentioned the UK Biobank (9 out of 21, or 42.8%). This is likely because the UK Biobank was undergoing its initial recruitment at this time (UK Biobank, 2014). While we could not determine from the data collected why a large number of articles were also published in 2012, reporting in that year emphasized the research related benefits of biobanking (*χ*^2^ = 352.576, *p* < 0.001) and featured quotes mainly from biobanking representatives (*χ*^2^ = 16.651, *p* > 0.001). Articles in that year were also mainly portrayed positively as promoting research and development (*χ*^2^ = 77.127, *p* > 0.001).

### General discussions of biobanking

A specific biobank is mentioned or identified in 123 or 75.5% of the articles included in the study. Among this subset of articles, the UK Biobank received the most mentions (in 61, or 49.6% of the articles), followed by Quebec’s CARTaGene (in 9, or 7.3% of the articles) (*χ*^2^ = 163.000, *p* < 0.001). Other identified biobanks received between 1 and 3 mentions. Majority of the articles that specifically mentioned the UK Biobank (58 out of 61) appeared in the UK-based newspapers included in our study. The remaining 3 mentions appeared in US newspapers (2) and an Australian newspaper (1) (*χ*^2^ = 289.320, *p* < 0.001).

Based on internet searches of the named biobanks, we categorized 38 as population or research biobanks and 9 as clinical biobanks. We were not able to determine the category or type for 6 of the named biobanks from the internet searches.

Locations of biobanks identified in the same subset of articles (*n* = 123) include the UK (in 69, or 56.1% of the articles), the US (in 18, or 14.6% of the articles), Canada (16 or 13% of the articles), and Australia (9 or 7.3% of the articles) (*χ*^2^ = 157.732, *p* < 0.001). Regarding the type of funding sources of the biobanks in the newspaper reports, 30 articles (18.4%) referenced public funding sources, 20 (12.3%) referenced private funding sources, and 32 (19.6%) mentioned or discussed a source representing public and private entities or interests. The most mentioned funding sources include the Medical Research Council (in 27, or 16.6% of articles), the UK’s Wellcome Trust (in 25, or 15.3% of articles) and the UK Department of Health (in 14, or 8.6% of articles). Mentions of these funding sources appeared primarily in UK based newspapers (*χ*^2^ = 124.444, *p* > 0.001).

Blood, tissue, DNA, urine and stem cells were the biological materials most commonly mentioned or discussed in the articles (61 or 37.4%, 44 or 27.0%, 40 or 24.5%, 22 or 13.5%, and 12 or 7.4% of the articles, respectively). Cancer was the most discussed disease type in the articles (in 58, or 35.6%), followed by diabetes (in 32, or 19.6%), cardiovascular disease (in 28, or 17.2%), Alzheimer’s disease (in 13, or 8.0%), Parkinson’s disease (in 12, or 7.4%), and mental health disorders including depression, bipolar disorder and post-traumatic stress disorder (in 12, or 7.4%).

Quotes about biobanking included in the articles were attributed to researchers in 77 articles (47.2%), biobank employees in 65 articles (39.9%), patients or donors in 23 articles (14.1%), government officials in 19 articles (11.7%), funding source representatives in 15 articles (9.2%), and clinicians in 14 articles (8.6%). Representatives of private industry were the least quoted source (in 9 articles, or 5.5%). Only a few articles (26, or 16%) featured a story about a patient or tissue donor.

Lastly, legal policy and regulatory matters were mentioned or discussed in only 64 articles (39.3%). The majority of these articles, totalling 41 articles, are clustered toward the beginning of the period analyzed (2002–2007), while the rest appear in articles published between 2008 and 2013 (*χ*^2^ = 21.710, *p* < 0.001).

### Benefits and risks

The majority of articles in the study sample mentioned or discussed the benefits of biobanking (137 or 84%), while fewer articles discussed risks (74 or 45.4%). Among the subset of articles that mention or discuss benefits, the main benefits discussed were related to medical research (115 or 83.9% of the articles) (*χ*^2^ = 148.434, *p* < 0.001). In the same subset, benefits were framed as promoting research and development in 117 articles (85.4%), as providing clinical or health benefits to patients receiving medical care in 16 articles (11.7%), as an economic benefit in 3 articles (2.2%), and as facilitating scientific progress and prestige in only 2 articles (1.4%) (*χ*^2^ = 155.605, *p* < 0.001). Among the most quoted categories of persons in the newspaper reports, researchers were quoted in slightly less than half of the articles that mention or discuss benefits (68, or 49.6% of the articles) (*χ*^2^ = 1.978, *p* > 0.001), while biobank employees were quoted in a fewer but significant number of articles within this subset (in 62 or 45.3% of the articles) (*χ*^2^ = 10.362, *p* = 0.001).

Among articles that mention or discuss risks (*n* = 74), privacy issues were identified as the main risk in 23 or 31% of the articles, followed by lack of research/general utility and scientific rationale (in 15, or 20% of the articles), funding and cost issues (in 6, or 8% of articles), and consent issues (in 5, or 6.7% of articles) (*χ*^2^ = 163.000, *p* < 0.001). More articles in this subset frame risks as ethics (39, or 52%) or research related (23, or 30.7%) than as clinical-related (6, or 8%) or economic (5, or 6.7%) (*χ*^2^ = 163.000, *p* < 0.001). Researchers were quoted in 36 or 48% of the articles that discuss risks (*χ*^2^ = 0.108, *p* > 0.001), while biobank employees were quoted in 27 or 36% of the same articles (*χ*^2^ = 0.650, *p* > 0.001).

Our raters scored mentions or discussions of certain benefits or risks that have received considerable attention in the academic and policy contexts, including the risk of discrimination in health insurance or other contexts, and health benefits for research participants or donors, including diagnostic and screening benefits. Only 12 articles (7.4%) of the entire study sample mentioned or discussed discrimination in the health insurance context, while 15 articles (9.2%) mentioned or discussed discrimination in other contexts. A similar number of articles (14, or 8.6%) mentioned or discussed health benefits directly accruing to research participants/donors, such as detection of disease, the communication to participants of clinical findings and potential clinical trials or research studies, and future therapeutic use of the samples provided, such as the cosmetic use of collagen provided from skin samples, the use of stem cells harvested from left over embryos from IVF treatments, and the potential use of banked umbilical cord blood and amniotic fluid cells.

### Portrayals/impressions of biobanking

Portrayals or impressions of biobanking in the articles included in our study were rated as mainly neutral (73 articles, or 44.8%) or positive (70 articles, or 42.9%). Fewer articles portrayed biobanking in a negative manner (20, or 12.3%). The dominant reason for the positive portrayals was linked to research and development (59 articles, or 36.2%), while ethical and research reasons accounted for the negative portrayals (10 articles, or 6.1%, and 9 articles, or 5.5%, respectively).

## Discussion

The portrayal of biobanking in the articles examined in this study was generally positive, with research-related benefits of biobanking prominently featured. This finding reinforces studies on media coverage of associated fields which, for example, have shown that the media often portrays developments in biotechnology and genetic research in an overly optimistic and simplistic manner (Conrad, 2001; Petersen, 2001; Bubela & Caulfield, 2004; Bubela et al., 2009). It is also not surprising that members of the research community were most often quoted in the articles analyzed in this study. It has been noted that researchers play a key role in providing information on scientific developments to the media, and in initiating or facilitating positive media portrayal of research outcomes (Caulfield, 2004; Bubela et al., 2009; Petersen, 2009). Our findings suggest that researchers play a role in regards to newspaper representations of benefits associated with biobanks, or at a minimum, contribute prominently to reporting trends. The overwhelming emphasis on benefits in our findings also strikes a sharp contrast with the underwhelming discussion of risks. This limited emphasis on risks is surprising considering that the social and scientific risks posed by biobanking, such as the issue of consent, have been a dominant source of controversy and debate in the academic and policy contexts (Master et al., 2012).

The prominent discussion of benefits in the majority of the articles analyzed also raises questions about the possible influence of newspaper reporting on donor or participant expectations. Studies have shown that research participants sometimes expect some form of personal benefit to accrue from their participation based on the mistaken belief that the purpose of the research study is to advance diagnostic or therapeutic interests rather than the actual purpose of creating generalizable knowledge (Clayton & Ross, 2006; Dixon-Woods et al., 2007; McCarty et al., 2007; Joseph et al., 2008; Haddow, 2009). While there is no basis from our findings to conclude that newspaper reporting on benefits of biobanks results in or contributes to “therapeutic or diagnostic misconception”, it may be worth investigating whether and to what degree such positive reporting shapes or influences participant views about expected outcomes from their participation in biobanking related activities.

Although only a few newspaper articles discussed clinical benefits to patients, it is significant that many of the articles contained discussions of different diseases that affect patients. This portrayal of biobanks being associated with several prominent diseases and, albeit to a lesser degree, with clinical benefits might have an influence on participants’ perceptions of biobanks. Indeed, a previous study has shown that various factors might influence an individual’s decision to participate in a population biobank study (Nobile et al., 2012), including individual dimensions such as personal predispositions (optimism, altruism, and trust) and subjective perceptions related to the study (perceived ease of participation, institution’s trustworthiness). Contextual dimensions such as family history of disease or external pressures have also been identified as playing a role in the decision process.

While one needs to be careful in inferring direct consequences between media discourses and participants’ views on biobanks, media representations can certainly have an impact on the different motives behind an individual’s decision to participate in research or not. Various studies have shown, for example, that the name and reputation of the institution that sets up the research projects predispose various participants to participate in research (Sinicrope et al., 2009; Lemke et al., 2010). If scandals reported in the media threaten the reputation of research institutions this will also threaten the potential participation of some individuals (Hoeyer, 2008).

Another noteworthy finding is that newspaper coverage is overwhelmingly focused on population or research biobanks. This finding echoes or reflects the increased attention, in the academic and policy realms, on issues, challenges and controversies associated with well-known population biobanking initiatives such as the UK Biobank and Iceland’s DeCODE (Barbour, 2003; Árnason, 2004; Tutton, Kaye & Hoeyer, 2004; Petersen, 2005; Knoppers & Abdul-Rahman, 2008; Hawkins, 2010). However, despite the increasing emphasis on regulatory and policy matters in the academic discourse, the finding that much of the newspaper reporting is clustered toward the beginning of the period analyzed suggests that media interest in such matters may be waning. While the reasons for this downtrend in interest are not clear from our study, a possible explanation is that the media views legal, regulatory or policy matters to be of lesser importance relative to the perceived benefits of biobanking, and therefore, not as interesting to discuss.

## Conclusion

Similar to media coverage of many forms of emerging biotechnology and associated research platforms, reporting on biobanks in the print media heavily favours discussions of related benefits over risks. The members of the scientific research community appear to be a primary source of this positive tone in reporting. The underreporting of risks, coupled with a downtrend in reporting on legal, policy and regulatory issues, suggest that the print media progressively views such matters to be less newsworthy or important relative to the perceived benefits of biobanking. Lastly, media coverage of biobanks appears to be overwhelmingly focused on notable population biobanks, including the UK Biobank and Quebec’s CARTaGene.

## Supplemental Information

10.7717/peerj.500/supp-1 Supplemental Information 1 Coding Frame

10.7717/peerj.500/supp-2 Supplemental Information 2 Data File

10.7717/peerj.500/supp-3 Appendix S1 Appendix A

10.7717/peerj.500/supp-4 Appendix S2 Appendix B
