# Supplementary material for: Newspaper coverage of biobanks
Source: PeerJ. 2014 Jul 31;2:e500. doi: 10.7717/peerj.500 (PMC4121587; doi:10.7717/peerj.500)
Supplement: Supplemental Information 1 [file peerj-02-500-s001.docx]

1. Country of newspaper
   1. Canada
   2. USA
   3. UK
   4. Australia
2. Newspaper
   1. The Globe and Mail
   2. National Post
   3. Toronto Star
   4. Montreal Gazette
   5. Vancouver Sun
   6. The Daily Telegraph (UK)
   7. Financial Times
   8. The Guardian
   9. The Times (London)
   10. The New York Times
   11. USA Today
   12. The Wall Street Journal
   13. The Washington Post
   14. Sydney Morning Herald
   15. The Age
   16. The Australian
3. Word count
4. Year of publication
5. Section of newspaper (exclude articles marked as “online only”)
   1. News
   2. Health/lifestyle/living
   3. Science
   4. Other
   5. Not specified
6. Article type
   1. News article
   2. Investigation or news analysis
   3. Editorial/opinion piece
   4. Letter to the editor
   5. Other
7. Author of article
   1. Reporter (health/science)
   2. Reporter (other than health/science)
   3. Expert commentary
   4. Mixed/multiple authors
   5. Not specified
   6. Other (including letters to editor)
8. Is a specific biobank mentioned?
   1. No
   2. Yes
9. If so, what biobank is mentioned? (free code)
   1. Not applicable
   2. CARTaGene
   3. Swedish Twin Registry
   4. UK BioBank
   5. Victorian Cancer Biobank
   6. Childhood Cancer and Blood Research (CCBR) BioBank
   7. Swedish National Biobank Program
   8. Canadian Prostate Cancer Network (CPC GENE) BioBank
   9. Newborn Screening Biobank (Denmark)
   10. The National Cohort (German)
   11. Children’s Hospital Boston Biobank
   12. DeCODE
   13. Denmark’s Biobank
   14. Kaiser Permanente's Biobank
   15. King’s College London Brain Bank
   16. UK Brain Bank for Autism
   17. Personal Genome Project
   18. Cell Care
   19. Donor Tissue Bank of Victoria
   20. Harvard Brain Tissue Resource Center
   21. UK Stem Cell Bank
   22. Military Brain Injury Studies Program
   23. Australian Breast Cancer Tissue Bank
   24. Melanoma Institute’s Tissue Bank
   25. Centre for the Study of Traumatic Encephalopathy at Boston University (“NFL’s brain bank”)
   26. Breast Cancer Campaign’s Biobank
   27. Quebec Brain Bank
   28. Vanderbilt Medical Center Gene Bank
   29. Stem Cell Bank at the Bernard O'Brien Institute of Microsurgery
   30. Parkinson’s Disease Society Tissue Bank
   31. Canadian Sports Concussion Project
   32. Multiple (West London Mental Health NHS Trust Brain Bank; University of California Brain Observatory, Biobank Japan, Estonian Genome Project, Western Australia Genome Health Project, Singapore Tissue Network, UK Biobank (x2), CARTaGENE, BC Biolibrary, Breakthrough Generations project)
   33. Genetic Alliance Biobank
   34. SeraCare Biobank
   35. GRAD Biobank
   36. CollaGenesis Biobank
   37. Sydney Cord Blood Bank
   38. MS Brain Bank
   39. Comprehensive Human Tissue Network
   40. Tumor Tissue Repository
   41. StemLifeLine Tissue Bank
   42. Alberta Cord Blood Bank
   43. Virgin Health Bank
   44. LifeBankUSA
   45. Stem Cell Resource
   46. Cryos International Sperm Bank
   47. Baker Heart Research Institute Gene Bank
10. Where is the biobank mentioned in the article located?
11. Not applicable
12. Canada
13. USA
14. UK
15. Australia
16. Other (specify; add separate code if 5 or more occurrences)
17. What, if any, is the primary biological material represented in the article?
    1. Blood
    2. Tissue
    3. Stem cells
    4. Tumors
    5. DNA
    6. Sperm
    7. Multiple (specify) (up to 3)
    8. Unspecified
    9. Urine
    10. Saliva
    11. Bone marrow
    12. Amniotic Fluid
    13. Embryos
    14. Cheek Swab
18. If mentioned, is the funding source for the biobank represented as public or private?
    1. Public
    2. Private
    3. Joint
    4. Unspecified
19. If a specific source of funding is mentioned in the article, what is it? (develop code)
    1. No source mentioned
    2. Multiple (specify) (up to 3)
    3. CN Miracle Match
    4. Lawrence Zimmering (charitable fundraising)
    5. US Government
    6. Asterand (a company)
    7. UK Department of Health
    8. PROCURE Foundation (non-profit)
    9. Wellcome Trust
    10. Kaiser Permanente
    11. Medical Research Council
    12. Multiple Myeloma Research Consortium
    13. Private Volunteers
    14. Cell care
    15. Australian Government
    16. “Open source” (anyone able to purchase)
    17. US Pentagon
    18. National Health and Medical Research Council [Australia]
    19. The National Football League
    20. The Fonds de recherche en santé du Quebec
    21. Parkinson’s Disease Society
    22. Government of Sweden
    23. International Science Foundations
    24. UK Government
    25. Charitable funding
    26. Prostate Cancer Canada
    27. Ontario Institute for Cancer Research
    28. Movember
    29. Helmholtz Association (Germany’s largest research organization)
    30. German federal government
    31. German state governments
    32. Children’s Hospital Boston
    33. Donations
    34. Genome Canada
    35. Genome Quebec
    36. Breast cancer campaign
    37. Asda’s Tickled Pink
    38. Walk the Walk Campaign
    39. Centre for the Study of Traumatic Encephalopathy at Boston
    40. Sports Legacy Institute (a non-profit)
    41. Multiple (UK Department of Health, the Scottish Executive, the Northwest, federal government, Quebec government, international partners
    42. Pioneer Fund
    43. CIHR
    44. Department of Health
    45. Carnegie Fund II
    46. BioLevier
    47. National Health Service
    48. MS Research Australia
    49. National Institutes of Health
    50. BC Cancer Agency
    51. AstraZeneca
    52. Glaxo SmithKline
    53. Virgin Group
    54. City of London
20. Does the article feature a patient/donor story?
    1. No
    2. Yes
21. What is (are) the condition(s)/disease(s) represented in the article? (develop code)
    1. No specific condition mentioned
    2. Mixed (specify as words)
    3. Cancer
    4. Schizophrenia
    5. Autism
    6. Dementia
    7. Diabetes
    8. Neurological disease
    9. Alzheimer’s
    10. Stroke
    11. Chronic Traumatic Encephalopathy (CTE)
    12. Staph infections (looking for genetic susceptibility to staph)
    13. Brain injury
    14. Parkinson’s
    15. Multiple Sclerosis
    16. Progeria
    17. ALS
    18. Chronic fatigue syndrome
22. Were any of the following quoted in the discussion of biobanking? 1 = no, 2 = yes
    1. Patient/donor
    2. Researcher
    3. Clinician
    4. Public/government official
    5. Biobank staff
    6. Funding official
    7. Private industry
23. Are benefits of biobanking mentioned in the article?
24. No
25. Yes
26. If so, how is the benefit framed? (Choose only the dominant frame.)
    1. Clinical (immediate health benefits)
    2. Research and development
    3. Economic
    4. Progress and prestige
    5. Other (specify)
    6. Not applicable

1. What is the main benefit discussed, if any?
   1. Not applicable
   2. Medical research
   3. Contributes to Innovation
   4. Useful for developing personalized medicine
   5. Useful clinical information for donor/patient
   6. Financial pay-offs/good financial investment
   7. Forefront of medical research and health care
   8. Contributes to economic growth
   9. Used in treatment/used in clinical trials
   10. Developing drugs and therapies
   11. Study of Disease
   12. Prevention/diagnosis/treatment of disease
   13. Screening for disease
   14. Prestige
   15. Better outcomes for transplantation
   16. Tissue donation
   17. Curing/eliminating disease
   18. Source for rare tissue
   19. Therapeutic transplantation
   20. Ethical source for stem cells
   21. Public source for stem cells
2. Does the article mention or discuss the following specific benefit or risk? (1 for no, 2 for yes):

a. Health benefits for research participants or donor, including diagnostic or screening benefits

b. Discrimination in health insurance

c. Discrimination in other contexts

1. Are risks or concerns regarding biobanking mentioned in the article?
   1. No
   2. Yes
2. If so, now are the risks or concerns framed?
   1. Clinical
   2. Ethical
   3. Economic
   4. Research
   5. Other (specify)
   6. Not applicable
3. What is the main risk or concern discussed, if any? (develop code)
   1. Not applicable
   2. Privacy
   3. Exploitation
   4. Not useful/ not effective/ won’t provide useful information
   5. Research without informed consent
   6. Bankruptcy (not economically viable)
   7. Discrimination in employment
   8. Lack of governance/standards/oversight
   9. Genetic discrimination
   10. Uncertainty about remit/application
   11. Patenting genes by companies
   12. Expensive
   13. Safety of product, need for safety testing
   14. Ethical issues associated with embryonic stem cells
   15. Lack of clear scientific rationale
   16. Ownership of tissue/research results
   17. Lack of research utility
   18. Lack of clinical utility
   19. Cost/funding
   20. Consent
   21. Diverts funding from other research
   22. Racial discrimination
   23. Sourcing of stem cells (abortion, etc.)
   24. Increases complexity of medical treatment
   25. Commodification
   26. Lack of control over what research is done with donated material
4. How is biobanking portrayed in the article as a whole?
   1. Neutrally
   2. Positively
   3. Negatively
5. If positive, why?
   1. Clinical
   2. Research and development
   3. Economic
   4. Progress and prestige
   5. Other (specify)
   6. Not applicable
6. If negative, why?
   1. Clinical
   2. Ethical
   3. Economic
   4. Research
   5. Other (specify)
   6. Not applicable
7. Are legal, policy, or regulatory issues relating to biobanking mentioned?
8. No
9. Yes
