# Supplementary material for: Newspaper coverage of biobanks
Source: PeerJ. 2014 Jul 31;2:e500. doi: 10.7717/peerj.500 (PMC4121587; doi:10.7717/peerj.500)
Supplement: Appendix S1 [file peerj-02-500-s003.docx]

**Appendix A**

| 1. Countries of origin of articles analyzed | | |
| --- | --- | --- |
| Country of newspaper | **Frequency** | **Percentage** |
| U.K | 85 | 52.1 |
| Canada | 30 | 18.4 |
| USA | 30 | 18.4 |
| Australia | 18 | 11.0 |

1. **Top newspapers, by name**

|  |  |  |
| --- | --- | --- |
| Newspaper name | **Frequency** | **Percentage** |
| The Guardian | 31 | 19.0 |
| Financial Times | 21 | 12.9 |
| The Times | 20 | 12.3 |
| The New York Times | 15 | 9.2 |
| The Montreal Gazette | 12 | 7.4 |

1. **Number of articles published, by year**
2. **Section of newspaper that the articles were located in**

|  |  |  |
| --- | --- | --- |
| Section of newspaper | **Frequency** | **Percentage of total articles** |
| News | 51 | 31.3 |
| Unspecified | 51 | 31.3 |
| Other | 41 | 25.2 |
| Science | 12 | 7.4 |
| Health or Lifestyle | 8 | 4.9 |

1. **Article type**

|  |  |  |
| --- | --- | --- |
| Article type | **Frequency** | **Percentage of total articles** |
| News | 78 | 47.9 |
| Investigative reports or news analysis | 53 | 32.5 |
| Opinion/Editorial | 14 | 8.6 |
| Other | 12 | 7.4 |
| Letters to editor | 6 | 3.7 |

1. **Type of author of articles**

|  |  |  |
| --- | --- | --- |
| Type of author | **Frequency** | **Percentage of total articles** |
| Health or science reporter | 89 | 54.6 |
| Reporter other than health/science reporters | 41 | 25.2 |
| Not specified | 15 | 9.2 |
| Expert commentators | 5 | 3.1 |
| Other (including letters to editor) | 7 | 4.3 |
| Mixed/multiple authors | 6 | 3.7 |

1. **Types of funding sources of biobanks mentioned in articles**

|  |  |  |
| --- | --- | --- |
| Type of funding source | **Frequency** | **Percentage of total articles** |
| Unspecified | 81 | 49.7 |
| Source representing public and private entities/interests | 32 | 19.6 |
| Public funding sources | 30 | 18.4 |
| Private funding sources | 20 | 12.3 |

1. **Top funding sources of biobanks mentioned in articles**

|  |  |  |
| --- | --- | --- |
| Funding source | **Frequency** | **Percentage of total articles** |
| No source mentioned | 91 | 55.8 |
| Medical Research Council | 27 | 16.6 |
| Wellcome Trust | 25 | 15.3 |
| UK Department of Health | 14 | 8.6 |

1. **Top biological materials mentioned in articles**

|  |  |  |
| --- | --- | --- |
| Biological material | **Frequency** | **Percentage of total articles** |
| Blood | 61 | 37.4 |
| Tissue | 44 | 27.0 |
| DNA | 40 | 24.5 |
| Urine | 22 | 13.5 |
| Stem cells | 12 | 7.4 |

1. **Top conditions/diseases mentioned in articles**

|  |  |  |
| --- | --- | --- |
| Condition/Disease | **Frequency** | **Percentage of total articles** |
| Cancer | 58 | 35.6 |
| Diabetes | 32 | 19.6 |
| Cardiovascular disease | 28 | 17.2 |
| Alzheimer’s disease | 13 | 8.0 |
| Parkinson’s disease | 12 | 7.4 |
| Mental health disorders | 12 | 7.4 |

1. **Type of people quoted in articles**

|  |  |  |
| --- | --- | --- |
| Type of person quoted | **Frequency** | **Percentage of total articles** |
| Researchers | 77 | 47.2 |
| Biobank employees | 65 | 39.9 |
| Patients or donors | 23 | 14.1 |
| Government officials | 19 | 11.7 |
| Funding source representatives | 15 | 9.2 |
| Clinicians | 14 | 8.6 |
| Representatives of private industry | 9 | 5.5 |

1. **Specific benefits and risks mentioned in articles**

|  |  |  |
| --- | --- | --- |
| Benefit or Risk | **Frequency** | **Percentage of total articles** |
| Discrimination in contexts other than health insurance | 15 | 9.2 |
| Health benefits directly accruing to research participants/donors | 14 | 8.6 |
| Discrimination in health insurance context | 12 | 7.4 |

1. **How biobanking was portrayed in articles**

|  |  |  |
| --- | --- | --- |
| Portrayal of biobanking | **Frequency** | **Percentage of total articles** |
| Neutrally | 73 | 44.8 |
| Positively | 70 | 42.9 |
| Negatively | 20 | 12.3 |
