# Supplementary material for: Newspaper coverage of biobanks
Source: PeerJ. 2014 Jul 31;2:e500. doi: 10.7717/peerj.500 (PMC4121587; doi:10.7717/peerj.500)
Supplement: Appendix S2 [file peerj-02-500-s004.docx]

1. Adam Cresswell, “Push to unlock melanoma genome secrets”, The Australian (29 August 2011) 3.
2. Alan Bavley, “Banking on the public, biologically; U.S seeks feedback on freezing samples from as many as 1 million people”, The Washington Post (13 April 2008) A7.
3. Alan Schwarz “Duerson’s brain trauma diagnosed”, The New York Times (3 May 2011) B11.
4. Alan Schwarz, “A chance for clues to brain injury in combat blasts”, The New York Times (23 June 2009) D5.
5. Alexandra Lopez-Pacheco, “Research hones in on new treatment; Canada helps hunt; ‘Life-changing results for cancer sufferers”, National Post (30 June 2011) FP10.
6. Alice Lighton, “Stem cells created with potential for human therapy, ‘Gold standard’ lines to enter clinical trials”, The [London] Times (7 December 2011) 23.
7. Alok Jha, “Critics’ u-turn as the world’s biggest medical project begins”, The Guardian (15 March 2006) 10.
8. Alok Jha, “From arthritis to diabetes: scientists unlock genetic secrets of diseases afflicting millions: Huge study covers seven common illnesses Hope of new cures for heart and bowel disorders”, The Guardian (7 June 2007).
9. Alok Jha, “Rory Collins: Blood, sweat and tears: Alok Jha meets the scientist who wants to recruit half a million people for a controversial medical project”, The Guardian (18 April 2006) 11.
10. Alok Jha, “Stem cell bank for drug testing may cut animal experiments”, The Guardian (3 October 2007).
11. Alok Jha, “World’s biggest medical experiment: Backstory”, The Guardian (23 February 2006) 15.
12. Alok Jha, “500,000 people, a span of decades - and a waste of time and money?: World’s biggest medical experiment: Huge study of causes of ill-health starts next week Project likely to turn into an albatross, say critics”, The Guardian (23 February 2006) 15.
13. Amelia Gentleman. “Inside the brain bank: Tomorrow is World Autism Day and new funding is in place to research the causes of the condition. But, writes Amelia Gentleman, the program now desperately needs tissue donors”, The Guardian (31 March 2013) 16.
14. Amy Corderoy, “Tens of thousands of lives saved in the running battle against cancers”, The Sydney Morning Herald (9 May 2013) 10.
15. Amy Corderoy, “World-first study raises hopes for children's quality of life”, The Age (5 December 2012) 1.
16. Amy Dockser Marcus, “Families hear gene secrets – Hospital study shifts protocol and will inform patients of worrisome test results”, The Wall Street Journal (9 June 2010) A3.
17. Amy Dockser Marcus, “Patients with rare diseases work to jump-start research – Advocacy groups create their own tissue banks to aid in drug development”, The Wall Street Journal (12 July 2006) D1.
18. Amy Dockser Marcus, “Aging fast: With just 42 known cases, drug trial is delicate task”, The Wall Street Journal (29 January 2007) A1.
19. Amy Harmon, “Where’d you go with my DNA?”, The New York Times (24 April 2010) WK1.
20. Andrea Stone, “Each stored embryo is a stem cell debate”, USA Today (30 January 2007) A1.
21. Andrew Braithwaite, Steve Brearton, Omar El Akkad, Ian Marlow & Nancy Won, “The world’s most creative cities”, The Globe and Mail (30 December 2011) 29.
22. Andrew Pollack, “DNA of blacks to be gathered to fight illness”, The New York Times (27 May 2003) A1.
23. Andrew Pollack, “Questioning the allure of putting cells in the bank”, The New York Times (29 January 2008) F1.
24. Antonio Regalado, “Plan to build children’s DNA database raises concerns”, The Wall Street Journal (7 June 2006) B1.
25. Arlene Klotzko, “Science matters”, FT Weekend Magazine (24 July 2004) 12.
26. Benedict Carey, “Brain banks for autism face dearth”, The New York Times (26 June 2012) D1.
27. Bernard Lane, “Overzealous guardians hamper research”, The Australian (16 August 2006).
28. Bill Pennington, “For athletes, the next fountain of youth?”, The New York Times (29 March 2007) D7.
29. “Boston Biomedics agrees to sell units to SeraCare Life”, The Wall Street Journal (19 April 2004) C4.
30. Brendan O’Keefe, “Chalmers for genome body”, The Australian (25 July 2007).
31. Bridie Smith, “First stem cell bank to open”, The Age (7 April 2009) 4.
32. Carolyn Abraham, “Stacking the deck; Would you make your DNA and health data public - if it may help cure disease? Canadian researchers are seeking volunteers, saying data on a mass scale is the only way to kick-start a stalled genetic revolution. But it could also be abused: Canada is the only G8 country with no law against genetic discrimination. In the first part of a two-week series exploring the social implications of genome research, Carolyn Abraham reports on an audacious experiment”, The Globe and Mail (8 December 2012) F1.
33. Charlene Sweeney, “O'Brien plan for stem-cell bank attacked”, The [London] Times (7 April 2008) 8.
34. Charlie Fidelman, “Project charts Quebec's genetic map; Tool for biomedical research; 20,000 people aged between 40 and 69 will be chosen at random to take part”, The Montreal Gazette (6 January 2010) A9.
35. Charlie Fidelman, “Quebec, Ottawa fund genetic mapping; Genome ‘biobank’ is one of the most ambitious undertakings to aid exchange of research info”, The Montreal Gazette (23 May 2007).
36. Charlie Fidelman, “Volunteers needed for genetic map; Would aid in medical research; 20,000 Quebecers sought to donate samples of blood and urine”, The Montreal Gazette (20 May 2009) A10.
37. Charlie Fidelman, “Waiting for the future of medicine; Doctors and searchers debate whether the promise of personalized treatment based on genetic data is being fulfilled”, The Montreal Gazette (11 October 2011) A4.
38. Charlie Fidelman, “We're sick and we just don't know it; genetic health project shows ‘huge portion’ of Quebec’s population has potentially serious health conditions”, The Montreal Gazette (15 January 2013) A6.
39. Christl Dabu, “ALS: Hope and anguish; Toronto researcher’s antibody marks an important breakthrough in battle against Lou Gehrig’s disease”, Toronto Star (4 September 2007).
40. Clara Pirani, “Business banking on cord blood”, The Australian (21 April 2007).
41. Clive Cookson, “A mission to research at the hospital bedside – Interview Prof John Bell – Not content to rest on his…”, Financial Times (18 July 2003) 13.
42. Clive Cookson, “Award for stem cell work shows benefit of collaborative research”, Financial Times (3 December 2012) 3.
43. Clive Cookson, “Biobank outlines its ethics for genetics study”, Financial Times (24 September 2003) 3.
44. Clive Cookson, “Biobank study to expand across country”, Financial Times (22 August 2006) 4.
45. Clive Cookson, “Gene bank”, Financial Times (15 December 2009), 10.
46. Clive Cookson, “**Gene research project wins £45m support” Financial Times (29 April 2002) 2.**
47. Clive Cookson, “Germany plans mass medical study”, Financial Times (4 July 2010) 4.
48. Clive Cookson, “Human genetics group plans to outlaw DNA theft”, Financial Times (21 May 2002) 3.
49. Clive Cookson, “Medical study recruits volunteers”, Financial Times (15 March 2006) 4.
50. Clive Cookson, “Inside track - Only businesslike visionaries need apply”, Financial Times (9 July 2002) 16.
51. Colin Blakemore, “Miracle cure or just a moral conundrum?”, The Daily Telegraph (3 February 2007).
52. David Adam, “Light at the end of the tunnel”, The Guardian (18 November 2004) 4.
53. David Dexter, “I want your brain, your health”, The [London] Times (30 August 2008) Body& Soul 8.
54. Deborah Smith, “News scan”, The Sydney Morning Herald (3 November 2011) 21.
55. “DNA collection”, The [London] Times (15 March 2006) 2.
56. “Society: Public Manager: Dream job?: Director of Communications, Biobank”, The Guardian (27 October 2004) 14.
57. Ed Pilkington, “The ultimate penalty: Before American football player Dave Duerson killed himself earlier this year, he asked that his brain be left to researchers studying head injuries among athletes. What it revealed shocked the scientists”, The Guardian (19 July 2011) 6.
58. “Genome project good for Quebec”, Editorial, The Montreal Gazette (9 January 2008) A20.
59. Geoff Dyer, David Firn, & Victoria Griffith, “Double helix is starting to make its mark in medicine”, Financial Times (4 July 2003) 20.
60. George Pendle, “Grey matters; Mind sciences – The science feature”, FT Magazine (25 November 2011) 44.
61. Gina Kolata, “Poking holes in genetic privacy”, The New York Times (16 June 2013) D3.
62. Gina Kolata, “Scientists unlock a mystery of staph”, The New York Times (15 December 2010) A24.
63. Gwen Kinkead, “To study disease, Britain plans a genetic census”, The New York Times (31 December 2002) 5.
64. Hannah Hoag, “Donations fill the bank, “This is the heart of the brain bank ,” Danielle Cecyre says, gesturing to the freezers and a pair of chrome shelving carts stacked with brains that have been donated to research”, The Montreal Gazette (15 January 2011) B4.
65. Hannah Hoag, “The brain and behaviour: How nurturing influences our minds; Epigenetics could help scientists understand why stress drives some to suicide or illness”, The Vancouver Sun (29 January 2011) C1.
66. Hayley Mick, “Unlocking clues to the mystery of aging”, The Globe and Mail (7 February 2008) L1.
67. Heather Walmsley, “The public has to be involved in debate on biobanking”, Editorial, The Vancouver Sun (9 October 2007).
68. Helena Kennedy, “Comments & Analysis - Bing’s genes concern us all”, The Guardian (22 May 2002) 17.
69. “Help for tomorrow”, Letter to Editor, The Daily Telegraph (16 March 2006) 21.
70. Hilary and Steven Rose, “Playing god – Eggs from fetuses, artificial wombs, dead men’s sperm – it’s not…”, The Guardian (3 July 2003) 25.
71. Hilary Rose, “Letter - DNA surveillance”, The Guardian (17 August 2012) 37.
72. Ian Sample, “500,000 medical records go online in public health database”, The Guardian (30 March 2012) 19.
73. Ian Sample, “Biotech firm offering DNA tests files for bankruptcy”, The Guardian (18 November 2009) 26.
74. Ian Sample, “Stem cell bank to begin supplying researchers: Pioneering UK facility to release embryonic cells, Center offers hope of medical breakthroughs”, The Guardian (18 September 2006).
75. James Meek, “Safeguards fail at gene bank says watchdog”, The Guardian, (14 January 2002) 8.
76. James Meikle, “Biggest gene bank seeks 500,000 volunteers – GP patients invited to take part but critics demand safeguards”, The Guardian (17 April 2002) 9.
77. James Randerson, “Autistic traits linked to testosterone in mother’s womb”, The Guardian (11 September 2007).
78. James Wilson, “Medical study to recruit 500,000”, Financial Times (18 April 2007) 2.
79. Jeanne Whalen, “An outcast among peers gains traction on Alzheimer’s cure”, The Wall Street Journal (10 November 2012) A1.
80. “Society: Public Manager: Job of the week”, The Guardian (18 June 2003) 14.
81. Jennifer Kingson, “An old Torah, older sunken boats and a seriously old primate”, The New York Times (10 June 2013) D2.
82. Jennifer Levitz, “Brain-Bank glitch hits research on autism”, The Wall Street Journal (12 June 2012) A5.
83. John Carvel, “Society: Comment: Red tape threatens last wish to aid brain research: Donated organs can boost understanding of conditions such as dementia, but those who want to give face many obstacles Brain donation hampered by red tape”, The Guardian (6 March 2012) 36.
84. John Carvel, “The NHS still has a way to go on brain donation”, The Guardian (28 May 2013) 35.
85. John Crace, “Education: Pennies from heaven: Alec Jeffreys tells John Crace how he stumbled on the process he later called genetic fingerprinting: Higher profile”, The Guardian (14 September 2004) 20.
86. Jon Wright, “World diary: May 26- Jun 1”, Financial Times (26 May 2008) 28.
87. Julia Medew, “Alfred boost for research scientists”, The Age (10 August 2012) 4.
88. Julie Rowbotham, “Victims give clues to the answers”, The Sydney Morning Herald (12 April 2007).
89. Julie Rowbotham and Linton Besser, “Rules break the skin and bone bank”, The Sydney Morning Herald (21 July 2012) 6.
90. Karen Deane, “Genetic data under fire”, The Australian (13 September 2005) 31.
91. Karen Deane, “Privacy alert on genetic database”, The Australian (12 September 2005) 5.
92. Kate Benson, “Banking on a new brain cure”, The Sydney Morning Herald (24 April 2008) 18.
93. Kate Wighton, “Science on a roll in 2007; News”, The [London] Times (30 December 2006).
94. Katherine Rowland and Andrew Jack, “Researchers gain access to data on 500,000 Britons; Health records”, Financial Times (30 March 2012) 4.
95. Kathryn Giusti, “Two wars against cancer”, The New York Times (29 December 2012) 7.
96. Kerry Gold, “Banking on the future; Today’s research to help tomorrow’s patients”, The Vancouver Sun (7 July 2012) E7.
97. Laura Johannes, “When your body needs spackle, it’s in the bank”, The Wall Street Journal (20 May 1998) NE2.
98. Laura Landro, “The informed patient: The growing clout of online patient groups”, The Wall Street Journal (14 June 2007) D1.
99. Lawrence Altman, “Virus is linked to a powerful skin cancer”, The New York Times (18 January 2008) A15.
100. “Loan boosts Galileo's search for bad genes”, The Montreal Gazette (20 January 2004) B5.
101. “Long aim for gene study”, The Guardian (7 April 2003) 14.
102. Margaret McCartney, “Second opinion”, FT Magazine (7 February 2009) 39.
103. Margaret Munro, “‘Biobanks’ seek deposits of untold value: your DNA: Scientists could make repeated withdrawals”, The Vancouver Sun (17 January 2005) A1.
104. Margaret Munro, “DNA debate heats up: Bioethicists say dilemmas created by biobanks defy easy resolution”, The Vancouver Sun (18 January 2005) A3.
105. Margaret Munro, “The brave new world of biobanking”, The Montreal Gazette (17 January 2005) A10.
106. Margaret Munro, “What can researchers do with your DNA?: Few biobanks are complying with existing consent rules, a lawyer says”, The Vancouver Sun (19 January 2005) A6.
107. Margot Date, “Give and let live”, The Sydney Morning Herald (22 May 2008) 14.
108. Mark Henderson, “Keeping it on record; Medical information; Junk medicine; News”, The [London] Times (30 June 2007).
109. Mark Henderson, “NHS staff fear burden of Virgin's stem-cell harvest”, The [London] Times (2 February 2007).
110. Mark Henderson, “Policing the gene pool; DNA fingerprinting; Junk Medicine; News”, The [London] Times (29 September 2007).
111. Mark Henderson, “Research funding wasted on useless projects, say MPs”, The [London] Times (25 March 2003) 13.
112. Mark Henderson, “Stem cell ‘insurance scheme’ exploits families, say scientists”, The [London] Times (16 October 2007).
113. Martin Mittelstaedt, “Low vitamin D in newborns linked to schizophrenia”, The Globe and Mail (10 September 2010) L1.
114. Mary Ormsby, “A CFL star’s brain and how it can help you; Former Toronto Argonaut Ted Toogood’s organ donation helps map links between neurological disease and concussions”, The Toronto Star (23 November 2012) A44.
115. Mary Ormsby, “The hurt locker (room) bond; Athletes, soldiers key focus behind concussion studies”, The Toronto Star (12 November 2011) IN3.
116. Melanie Reid, “Half a million volunteers to take part in world's biggest health study”, The [London] Times (17 July 2007).
117. Michael Laris, “In a Va. lab, forging links to speed cancer advances; GMU, with ties to Italy, aims to be biotech force”, The Washington Post (27 February 2008) B1.
118. Mike Wade, “Stem cell treatment that offers hope to stroke victims passes first clinical trials”, The [London] Times (2 September 2011) 14.
119. Natasha Wallace, “Bank puts a stop to brain drain”, The Sydney Morning Herald (6 February 2008) 7.
120. Nicholas Wade, “Hunting for disease genes in Iceland’s genealogies”, The New York Times (18 June 2002) 4.
121. Nick Collins, “‘Gold standard’ stem could lead wave of treatments for diseases”, The Montreal Gazette (7 December 2011) A15.
122. Nick Hassell Tempus, “Asterand”, The [London] Times (31 March 2009) 47.
123. Nikki Tait, “EU law could assist ‘biobanks’”, Financial Times (17 September 2009) 2.
124. “Organ donors’ wishes and the views of surviving relatives”, Letters to the Editor, The [London] Times (15 August 2012) 21.
125. Pamela Fayerman, “A decade of success; B.C. now has some of the best treatment outcomes in the country for many types of cancer, thanks in large part to the innovative work at the BC Cancer Agency”, The Vancouver Sun (27 October 2007).
126. Paul Waldie, “Setting the pace for cancer research; The Donor: Lawrence Zimmering”, The Globe and Mail (16 June 2012) B2.
127. Polly Curtis, “Banking on the future – Sir George Radda tells Polly Curtis about the…”, The Guardian (19 March 2002) 35.
128. Rebecca Skloot, “Taking the least of you”, The New York Times (16 April 2006) 38.
129. Rhonda Rundle, “Kaiser seeks genetic data in effort to build database”, The Wall Street Journal (15 February 2007) B3.
130. Richard Irving, “UK charity leads way with move into bond market”, The [London] Times (4 July 2006) 45.
131. Rick Weiss, “Scientists see potential in amniotic stem cells; They are highly versatile and readily available”, The Washington Post (8 January 2007).
132. Roger Highfield, “£British Association 62m Biobank may not be worth it, says professor”, The Daily Telegraph (6 September 2004) 8.
133. Roger Highfield, “500,000 to provide DNA samples for ‘Biobank’”, The Daily Telegraph (29 April 2002) 6.
134. Roger Highfield, “Wanted: Half a million medical guinea pigs”, The Daily Telegraph (15 March 2006) 9.
135. Roger Highfield, “In Brief Roger Highfield on tissue banking, defeating BSE and new kinds of ice”, The Daily Telegraph (4 April 2006) 27.
136. Roger Highfield, “Leader of DNA bank defends project”, The Daily Telegraph (7 April 2003) 13.
137. Roger Highfield, “Science briefs”, The Daily Telegraph (4 August 2004) 14.
138. Roger Highfield, “The politics of stem cell ethics”, The Daily Telegraph (9 January 2007).
139. Roger Highfield, “Today, we’d ignore Einstein Original thinking is being strangled by bureaucracy…”, The Daily Telegraph (1 October 2003) 17
140. Rory Collins ,“Letter: Adios or arrivederci?”, The Guardian (17 August 2012) 37.
141. Rory Collins, “What UK Biobank is - and is not”, Letters to Editor, Financial Times (4 September 2007) 12.
142. Salamander Davoudi, “Gene survey aims to assess risk of disease”, Financial Times (29 August 2007) 4.
143. Sarah Boseley, “Autism study: Disorder linked to high levels of testosterone in the womb: Prenatal screening tests could follow ground breaking research into 235 children”, The Guardian, (12 January 2009) 6.
144. Sarah Boseley, “Privacy fears may slow big genome project to tackle killer diseases: Only one in 10 sign up after invitation to project: UK biobank chief defends biobank security measures”, The Guardian (30 December 2008) 16.
145. Sarah Danckert, “In Brief”, The Australian (8 March 2012) 30.
146. Sarah Hall, “£61m medical experiment begins”, The Guardian (22 August 2006) 7.
147. Sheila McGovern, “Galileo invests in genes: $11.2 million raised. Looking for cures for several diseases”, The Montreal Gazette (8 January 2004) B3.
148. Sheryl Ubelacker, “Banking your baby's blood; Some parents are storing regenerative stem cells found in their child's umbilical cord. But experts say their fears are largely unfounded”, The Globe and Mail (4 May 2007).
149. Shirley English, “World's biggest medical study seeks volunteers”, The Times (London) (6 June 2007).
150. Shirley Wang, “Doubling up on research using a database of twins”, The Wall Street Journal (5 November 2012) D1.
151. Simon Crompton, “Banking on the future for breast cancer”, The [London] Times (8 March 2011) 7.
152. Steve Jones, “View from the lab: Biology, bureaucracy, banks and big bucks”, The Daily Telegraph (1 October 2003) 17.
153. Steven Poole, “Stone-age mind? Speak for yourself: Steven Poole praises a strong expose of the hype surrounding genetics and neurosciences: Genes, cells and brains: The Promethean Promise of the New Biology by Hilary Rose and Steven Rose”, The Guardian (1 December 2012) 7.
154. Tenille Bonoguore, “Science city; Tenille Bonoguore reports on our booming medical research scene and meets 12 of the best specimens. Photographer Tory Zimmerman heads out to capture them in their natural habitats”, The Globe and Mail (5 January 2008) M1.
155. Thomas Stuttaford, “The quest to find the key to physical and mental illness”, The [London] Times (31 October 2005) 13.
156. Tim Radford, “Discovering how to read the book of life”, The Guardian (14 April 2003) 8.
157. Tim Radford, “Gene study will provide 30 years of human data- Manchester-based UK Biobank to monitor health of…”, The Guardian (24 September 2003) 9.
158. “Universities to tackle prostate-cancer mystery”, The Montreal Gazette (28 October 2008) A6.
159. “Value of patient data”, Letter to the Editor, The Daily Telegraph (10 December 2011) 27.
160. “Vital health research suffers as UK funds frittered”, The Australian (9 April 2003) 25.
161. Vivienne Parry, “Used in evidence; Stories behind the news, The [London] Times (2 August 2008) Body & Soul 2.
162. Will Pavia, “The Vikings are coming”, The Times (London) (27 November 2006).
163. William Hall, “Germ of an idea takes root Pharmaceuticals and Biotechnology: A spate of pump pricing has boosted the sector’s prospects, writes William Hall”, Financial Times (26 April 2007) 4.
